# Обзор и комментарии к «Руководству по обогащению соли йодом: подтвержденная приверженность достижению оптимального уровня йода в питании»

**DOI:** 10.14341/probl13717

**Published:** 2026-07-22

**Authors:** Г. А. Герасимов

**Affiliations:** Международная неправительственная организация «Глобальная сеть по йоду» Соединённые Штаты Америки; Internationl NGO “Iodine Global Network” United States

**Keywords:** йодированная соль, йодный статус, беременные женщины, йод в моче, дозорный эпидемилогический надзор, iodized salt, iodine status, pregnant women, urinary iodine, sentinel epidemiological surveillance

## Abstract

За первую четверть ХХI века йодированная соль стала доступна практически повсеместно, и в мире сохраняются лишь отдельные очаги йодного дефицита. К 2024 г. 126 стран мира ввели обязательное обогащение соли йодом, 89% жителей планеты потребляют йодированную соль, а 101 страна достигла адекватного йодного статуса населения, тогда как в 1993 г. низкое потребление йода имело место в 113 странах. Именно поэтому обогащение соли йодом признано одним из величайших достижений общественного здравоохранения. Новые рекомендации, рассмотренные в данном обзоре, обновляют существующие руководства, регламентирующих программы обогащения соли, их мониторинг и оценку, и предлагают использовать новые подходы в сочетании с сохранением и укреплением мер, доказавших свою эффективность и устойчивость. Из 6 «аналитических записок», сходящих в руководство, наибольший интерес для читателей может представлять раздел, посвященный повышению доступности данных о йодном статусе населения. По мнению авторов, оценка эффективности программ йодирования соли должна осуществляться путем интеграции обследований йодного статуса в более комплексные обследования здоровья и питания населения либо посредством дозорного эпидемиологического мониторинга в районах высокого риска йодного дефицита. Сбор образцов мочи в женских консультациях при рутинном обращении в них беременных женщин, вероятно, будет менее ресурсоемким подходом, чем репрезентативные поперечные обследования йодного статуса.

## Abstract (en)

During the first quarter of the 21st century, iodized salt became available almost everywhere, and only isolated pockets of iodine deficiency remain in the world. By 2024, 126 countries had introduced mandatory iodine fortification of salt, 89% of the world’s population consumes iodized salt, and 101 countries have achieved adequate iodine status among their populations, whereas in 1993, low iodine intake was observed in 113 countries. This is why iodine fortification of salt is recognized as one of the greatest achievements in public health. The new recommendations reviewed in this paper update existing guidelines regulating salt fortification programs, their monitoring and evaluation, and propose using new approaches in combination with maintaining and strengthening measures that have proven effective and sustainable. Of the six "analytical notes" included in the guidelines, the section on improving the availability of data on the iodine status of the population may be of greatest interest to readers. According to the authors, the effectiveness of salt iodization programs should be assessed by integrating iodine status surveys into more comprehensive health and nutrition surveys of the population or through sentinel epidemiological monitoring in areas at high risk of iodine deficiency. Collecting urine samples in antenatal clinics during routine visits by pregnant women is likely to be a less resource-intensive approach than representative cross-sectional surveys of iodine status.

## Введение

Обогащение соли йодом для профилактики йодного дефицита и его последствий считается глобальным успехом общественного здравоохранения [1]. Сегодня соль, обогащенная йодом, доступна практически повсеместно, и в мире сохраняются лишь отдельные очаги йодного дефицита. К 2024 г. 126 стран мира ввели обязательное обогащение соли йодом, а еще 22 страны разрешают йодировать соль на добровольной основе. Обогащение соли йодом является наиболее распространенной формой крупномасштабного обогащения пищевых продуктов (КОПП) в современном мире [2].

В результате этих усилий 89% жителей планеты потребляют йодированную соль [3], а обеспеченность питания йодом значительно улучшилась повсеместно [1]. Первая оценка ВОЗ глобальной распространенности эндемического зоба в 1960 г. показала, что им страдало 20–60% населения мира, причем большая часть бремени приходилась на страны с низким и средним уровнем дохода [4]. Нынче из 127 стран, имеющих национальные данные, 101 страна имеет адекватный йодный статус населения и только 18 — недостаточный, тогда как в 1993 г. низкое потребление йода имело место в 113 странах [5]. Именно поэтому обогащение соли йодом признано одним из величайших достижений общественного здравоохранения [1].

В определенной мере успех программ йодирования соли был связан с технической и финансовой помощью странам со стороны донорских организаций и координацией усилий со стороны Всемирной организации здравоохранения (ВОЗ), Детского Фонда ООН (ЮНИСЕФ), Глобальной сети по йоду (ГСЙ)1 и других международных, правительственных и неправительственных организаций. Так, ВОЗ в сотрудничестве с ЮНИСЕФ и ГСЙ в 2007 г. выпустили руководство по обследованию и мониторингу йододефицитных заболеваний (ЙДЗ), предназначенное для руководителей национальных программ йодной профилактики [6]. К сожалению, это официальное руководство не обновлялось уже почти 20 лет. В какой-то мере отсутствие основного руководящего документа ВОЗ компенсировалось выпущенным в 2014 г. руководством ВОЗ по нормативам йодирования соли [7] и рекомендациями ЮНИСЕФ (2018), внесшими коррективы в нормативы мониторинга дефицита йода [8].

Несмотря на значительные достижения последних двух десятилетий, в некоторых странах сохраняется дефицит йода, а в ряде имеется его избыточное потребление. Изменения в типах потребления соли выдвинули на первый план новые проблемы, и устойчивость некоторых национальных стратегий профилактики может оказаться недостаточной. Так, относительно мало внимания уделялось обеспечению использования йодированной соли при производстве пищевых продуктов промышленной переработки (ППП), несмотря на растущее число стран, в которых население потребляет основную часть соли именно с ними [9]. Среди международных организаций, поддерживающих обогащение соли, отсутствует консенсус относительно того, как следует осуществлять и контролировать обогащение соли йодом, и на каких основных мерах следует сосредоточиться, например, в отношении поддержки мелких производителей [10].

Обогащение соли также часто воспринимается как мера, противоречащая усилиям по сокращению потребления соли, что приводит к снижению политической поддержки [11]. Основной индикатор — медианная концентрация йода в моче (мКЙМ), отражающая йодный статус, часто неверно интерпретируется, что приводит к неправильным оценкам и действиям. Кроме того, ряд используемых индикаторов, включая долю домохозяйств, использующих адекватно йодированную соль, не фокусирует внимание на обогащении соли для ПППП, которое требует повышенного внимания [8]. Успех обогащения соли йодом в некоторых странах привел к попыткам добавлять в соль дополнительные микронутриенты, что потенциально наносит ущерб текущим мерам по йодированию соли [12].

Необходимость в обновлении руководства по обогащению соли обусловлена пониманием того, что принцип «больше того же самого» не решит проблемы, обозначенные выше. Продолжение внедрения йодирования соли в том же ключе, что и в прошлом, не приведет к более широкому охвату населения обогащенной солью или оптимальному йодному статусу у всего населения. Необходимы новые подходы к обогащению соли в сочетании с сохранением и укреплением мер, доказавших свою эффективность и устойчивость.

В этой связи ЮНИСЕФ в сотрудничестве с Фондом Гейтса, Глобальным альянсом за улучшение питания, Глобальной сетью по йоду, центрами США по контролю и профилактике заболеваний и ВОЗ подготовили новое руководстве по обогащению соли йодом [2], содержащее 6 аналитических записок, для совершенствования мер по достижению адекватного и устойчивого уровня потребления йода в мире.

Поскольку данное руководство, опубликованнное только на английском языке, является довольно объемным, в настоящей статье дается сокращенный обзор отдельных аналитических записок, входящих в этот документ, сопровождаемый собственными комментариями автора.

Первый из этих комментариев относится к используемой терминологии: в обсуждаемом руководстве в основном используется термин «обогащение2 соли» или «обогащение соли йодом», а не «йодирование соли» или «всеобщее йодирование соли». Термин «йодирование соли» если и используется, то в основном в тех случаях, когда необходимо разграничить обогащение соли йодом и обогащение соли другими микронутриентами. С точки зрения авторов аналитических записок, это позволяет рассматривать обогащение соли йодом в более широких и устойчивых рамках и способствует достижению глобального консенсуса по вопросу интеграции йодирования соли в более широкую концепцию крупномасштабного обогащения пищевых продуктов (КОПП).

## Аналитическая записка №1: Переориентация обогащения соли в новых условиях

Рекомендации этой записки, можно сравнить с увертюрой к опере, в которой присутствуют основные музыкальные темы всего произведения, и вкратце сводятся к тому, что следует повышать осведомленность лиц, формирующих политику и принимающих решения в области здравоохранения, о широкой распространенности и негативном влиянии легкого дефицита йода на показатели здоровья, а также о важности постоянного обогащения соли йодом. Необходимым условием повышения осведомленности о дефиците йода является доступность актуальных данных о йодном статусе населения. В аналитической записке 5 обсуждаются некоторые способы достижения этой цели. Также считается полезным признать йодирование соли в качестве формы крупномасштабного обогащения пищевых продуктов, что потенциально может улучшить реализацию и повысит устойчивость стратегии йодирования соли. Конкретные предложения по достижению этой цели обсуждаются в аналитической записке 2. Также следует создать политическую и нормативно-правовой среду, гарантирующую, что вся пищевая соль (столовая соль и используемая в пищевой промышленности) обогащается йодом. Это можно достигнуть путем принятия законодательства об обязательном обогащении пищевых продуктов. Мировой опыт показывает, что масштабы обогащения соли выше в странах с законодательством об обязательном обогащении, чем в странах, где оно проводится на добровольной основе. В аналитической записке 4 обсуждаются методы обеспечения соблюдения национальных требований к обогащению соли.

## Аналитическая записка №2: Интеграция соли в программы обогащения других пищевых продуктов и пищевые системы3

По мнению авторов этой записки, одной из возможных причин стагнации и спада масштабов обогащению соли в ряде стран является то, что они реализуются как самостоятельная (не интегрированная) программа. Это означает, что, хотя обогащение соли предполагает добавление обогащающего вещества (например, йодата калия) к регулярно потребляемому пищевому продукту (соли), оно не признается формой КОПП и не внедряется в рамках существующих мер по исполнению законодательства в области пищевых продуктов. Кроме того, в странах, где другие массовые пищевые продукты (в первую очередь пшеничная мука) подлежат обязательному обогащению, йодирование соли часто регулируется иным образом, чем обогащение остальных продуктов. Например, нередко бывает так, что один закон касается обогащения соли, а другой — всех остальных продуктов, или же обогащение солью контролируется иным государственным ведомством, чем другие обогащенные продукты.

Причиной этого может может быть то, что соль была первым пищевым продуктом, который начали обогащать в широком масштабе в начале 1920-х годов в качестве средства профилактики дефицита йода. Таким образом, предшествующего опыта, на который можно было бы опереться, еще не было, и обогащение соли часто разрабатывалось и внедрялось как самостоятельная программа общественного здравоохранения, а не в рамках интегрированного подхода на основе пищевых систем. Но поскольку соль является приправой, а не продуктом питания, она часто не принимается во внимание при рассмотрении вопроса об обогащении основных пищевых продуктов.

Тут требуется пояснение: нынче под КОПП понимается обогащение микронутриентами массовых пищевых продуктов — таких, как пшеничная и кукурузная мука, растительное масло или молоко. В настоящее время в более чем 90 странах мира ежегодно производят десятки миллионов тонн обогащенной муки. В десятках стран, включая США, Канаду, большинство стран Латинской Америки, многие страны Азии и Африки, обогащение муки микронутриентами (железом, фолиевой кислотой, цинком, витаминами группы B) является обязательным. Примерно в 30 странах витаминами А и Д в массовом масштабе обогащаются растительное масло и маргарин, а в молоко добавляется витамин Д и кальций. В некоторых азиатских странах, где потребление зерновых продуктов и молока невелико, проводится обогащение витаминами риса и сахара [13].

Но то, что может быть устоявшейся нормой в одних странах, является скорее исключением в других. Так, в большинстве европейских стран муку микронутриентами практически не обогащают, а растительное масло и молоко с витаминами производятся в ограниченном количестве. На уровне Европейского Союза имеются нормативные документы, определяющие перечень веществ, которые разрешено использовать для обогащения соли, муки и других пищевых продуктов. Также регулируется ряд других аспектов обогащения пищевых продуктов, включая обязательные требования к информации на упаковке. Однако все остальные вопросы, включая обязательные или добровольные требования к обогащению соли и других пищевых продуктов, тип обогащающего вещества и его количество, определяется правительствами отдельных стран. В России также не существует сколь-либо масштабного обогащения пищевых продуктов (кроме соли), а если такие продукты и выпускаются в незначительных объемах, то обогащение используется скорее в целях их рекламы и продвижения. К сожалению, для стран, где крупномасштабное обогащение пищевых продуктов фактически отсутствует, рекомендации аналитической записки 2 не имеют практичского значения.

Но в целом, приравнивание йодирования соли к обогащению других массовых пищевых продуктов является правильным решением, поскольку включает йодированную соль (которая в пищевом смысле является приправой) в число других «полезных» пищевых продуктов. Дискриминировать йодированную соль как «вредный» продукт станет сложнее.

## Аналитическая записка №3: Поддержка обогащения соли мелкими производителями

Сразу оговорюсь: для России и большинства европейских стран содержание этой записки мало релевантно, но в этом случает — не к сожалению, а скорее к счастью, так как крупномасштабная добыча, переработка, обогащение йодом и последующая упаковка соли производится на предприятиях, имеющих адекватную технологию производства и системы управления и контроля качества продукции. Иными словами, они созданы, чтобы выпускать качественную соль с адекватным содержанием йода. В то же время многолетний мировой опыт показывает, что малые и кустарные производители качественную йодированную соль выпускать не могут, да часто и не хотят. Даже если им для этого бесплатно предоставляется все нужное для йодирования соли (оборудование, йодат калия, средства контроля качества), то они при первой же возможности прекращают это безнадежное дело, а если на них наседает государство, то лишь чуть-чуть набрызгивают раствор йодата калия на соль, чтобы при тестировании крахмальным раствором она окрашивалась в синий цвет. В нашем регионе проблема малых производителей соли актуальна для Таджикистана и Узбекистана. Самое адекватное решение проблемы — консолидация производства йодированной соли на небольшом числе достаточно крупных и хорошо оснащенных предприятий. Большинство стран Восточной Европы и Центральной Азии либо уже имеют крупных производителей, обладающих современными системы управления качеством продукции, либо импортируют качественную йодированную соль из соседних стран [13].

Однако в целом ряде более бедных стран проблема низкого качества обогащения соли йодом остается актуальной, а эффективные меры их решения по разным причинам остаются недоступными. По мнению авторов этой аналитической записки, глобальный урок заключается в том, что устойчивое обогащение продукции мелкими производителями соли может быть достигнуто только при условии их сотрудничества в целях создания жизнеспособной бизнес-модели, позволяющей им прибыльно производить соль, соответствующую национальным стандартам, и при условии их включения в эффективные государственные системы надзора, обеспечивающие равные условия для всех производителей соли, как крупных, так и мелких.

## Аналитическая записка №4: Совершенствование методов оценки качества обогащения соли

В самой сжатой форме суть предложения экспертов состоит в использовании для внутреннего и внешнего контроля качества композитных (составных) образцов йодированной соли вместо разовых проб. Процесс обогащения соли йодом требует равномерного распределения одной части обогащающего вещества (йодата калия) на 20 тысяч частей соли (представьте себе равномерное перемешивание небольшого числа иголок в стоге сена). Это непростая задача, но современные и технически оснащенные предприятия по производству соли с ней вполне справляются. Проблема в более мелких и слабо оснащенных фабриках, где возможны значительные колебания в уровне йода в отдельных пробах соли. Для устранения этих вариаций предлагается смешивать разовые образцы соли, которые регулярно собирают с конвейера в течение смены, в большой композитный образец и, тщательно перемешав, определять в нем среднюю концентрацию йода. Так, кстати, уже поступают на большинстве мукомольных предприятий для контроля обогащения продукции витаминами и микроэлементами4. Сходным образом можно поступать с отдельными образцами соли, собранными в домохозяйствах. Помимо снижения вариабельности в уровне йода, определенную экономию можно получить за счет снижении объема лабораторной работы.

Не думаю, что эта рекомендация актуальна там, где нет проблем ни с качеством йодированной соли, ни с методами ее оценки.

Авторы аналитической записки обращают внимание на то, что во многих странах нормативное содержание йода устанавливается не как вариация вокруг среднего уровня, а как диапазон или даже в виде минимальной или максимальной концентрации. По данным Global Fortification Data Exchange, из 136 известных стандартов пищевой соли, допустимое содержание йода выражено как максимальный предел в 4 странах, минимальный уровень — в 20 странах и диапазон концентрации — в 87 странах. Только 25 стран указывают нормативное содержание йода в качестве среднего показателя с допустимыми отклонениями выше и ниже целевого значения [15]. Если среднее значение йода в соли не указано, то производители соли нередко добавляют только минимальное количество йода в соль (нижнюю границу допустимого диапазона), в то время как органы надзора за качеством пищевых продуктов рассматривают такую соль как несоответствующую требованиям, если содержание йода ниже минимально допустимого или выходит за пределы допустимого диапазона. Таким образом, экономия на йодате калия может обернуться для производителя крупными штрафами.

## Аналитическая записка №5: Повышение доступности данных о йодном статусе населения

Это аналитическая записка является, на мой взгляд, наиболее интересной и актуальной для читателей и излагается в этом обзоре максимально подробно. Согласно текущим рекомендациям [6][16], йодный статус наилучшим образом оценивается путем измерения медианной концентрации йода в моче (мКЙМ) в популяции. Если мКЙМ у детей школьного возраста или взрослых составляет 100 мкг/л или более, считается, что во всей исследуемой группе населения имеется адекватное потребление йода [6].

**Table:** *Более поздние рекомендации ЮНИСЕФ [8] расширили диапазон оптимальной мКЙМ до 100–300 мкг/л.

Выдержка из рекомендаций ВОЗ, ЮНИСЕФ и Глобальной сети по йоду [6] по мониторингу устранения йододефицитных заболеваний по показателям йодного статуса населения• Медианная КЙМ у детей школьного возраста должна быть в диапазоне от 100 до 199 мкг/л*• Медианная КЙМ у беременных женщин в популяции должна быть в диапазоне от 150 до 250 мкг/л• Данные по йодному статусу (на национальном или региональном уровне) должны быть получены в течение последних 5 лет

Однако во многих странах в настоящее время отсутствуют недавние сведения о йодном статусе населения, что может отражать снижение интереса к обогащению соли, нехватку финансирования для обследований йодного статуса населения или ошибочное представление о том, что дефицит йода больше не представляет риска для общественного здоровья. Еще одна проблема заключается в том, что даже при наличии данных о йодном статусе на национальном уровне такая информация может быть не доступна на региональном (субнациональном) уровне, а это означает, что в стране могут существовать не выявленные очаги дефицита йода.

По данным «Системы информации ВОЗ5 по витаминам и минералам» (VIMNS) [17], только 10 стран располагают данными о йодном статусе любой группы населения за последние четыре года (2020–2024 гг.), а 95 стран имеют данные за последние 10 лет (2014–2024 гг.). Из 95 стран, имеющих сведениями за последние 10 лет, 68 располагают данными как минимум до первого административного уровня (область, провинция и т.п.). Таким образом, 51% стран вовсе не имеют информации о йодном статусе населения за последние 10 лет, а для 65% стран нет данных о йодным статусе на субнациональном уровне. В связи с этим большинство стран не могут оценить текущее влияние обогащения соли йодом на йодный статус и, следовательно, не способны выявить проблемы и принять меры по их устранению, таких как корректировка содержания йода или целевые программы для конкретных географических регионов.

Большинство (53%) данных о йодном статусе были получены из национальных репрезентативных поперечных исследований, некоторые из которых также проводили оценку на субнациональном (региональном) уровнях, в то время как 12% данных были получены только из субнациональных обследований, а национальные данные остались недоступными [17]. Только 28% данных о йодном статусе было получено из комплексных исследований здоровья, питания или потребления других микронутриентов, а также исследование STEPS [18] на Украине.

Эта информация указывает на то, что большинство данных о йодном статусе были получены из специальных национальных или субнациональных обследований, и только треть — из более широких обследований или систем надзора за здоровьем и питанием.

Йодный статус может быть оценен более простыми и дешевыми методами по сравнению с репрезентативными поперечными обследованиями. Хотя такие обследования, безусловно, являются важным источником информации о йодном статусе, они требуют больших затрат. Нехватка новых данных, вероятно, является результатом сокращения внешнего финансирования проектов по устранению йодного дефицита. Более того, опора на репрезентативные йодные обследования не способствует интеграции йода в более широкие программы по улучшению питания и может негативно влиять на устойчивость обогащения соли. Было доказано, что оценка йодного статуса может быть интегрирована в комплексные обследования состояния здоровья, питания или обеспеченности микронутриентами. Включение оценок йодного статуса в более широкие обследования или системы наблюдения требует меньше ресурсов, чем отдельные обследования йодного статуса.

Дозорный эпидемиологический надзор6 (ДЭН) позволяет оценить йодный статус в районах высокого риска. В то время как национальные репрезентативные обследования являются золотым стандартом для получения достоверных данных о йодном статусе населения, ДЭН предлагает альтернативный подход, позволяющий быстрее и экономичнее собирать данные о потреблении йодированной соли и йодном статусе. ДЭН — это надзор, проводимый на конкретных дозорных участках или среди конкретных групп населения, а не по всей стране или среди всего населения. Хотя ДЭН не может полностью заменить национальные репрезентативные обследования, он повышает эффективность сбора данных, предоставляя более регулярную информацию для оптимизации профилактических программ. Система ДЭН может быть создана в стратегически важных районах для мониторинга йодного статуса. Дозорные участки могут располагаться в районах с повышенным риском дефицита йода, например, в сельской местности, в районах с недостаточным охватом населения обогащенной солью, в высокогорных районах или в регионах со значительным финансовым неравенством населения. На дозорных участках образцы мочи могут собираться в школах или женских консультациях для облегчения доступа к ключевым группам населения.

Женские консультации (центры дородового наблюдения) предоставляют возможность оценить йодный статус у беременных женщин — важной целевой группе населения. В странах с высокой посещаемостью женских консультаций оценка мКЙМ у беременных женщин может предоставить данные о йодном статусе в этой целевой группе населения, для которой проводится обогащение соли. Центры дородового наблюдения обычно собирают образцы мочи у всех беременных для выявления инфекций мочевого пузыря или почек, диабета и преэклампсии. Определение КЙМ можно проводить в уже собранных пробах мочи, что позволит существенно сократить логистические расходы. Данные мКЙМ, полученные в женских консультациях, помогут оценить долю беременных женщин с адекватным йодным статусом и потенциальную обеспеченность йодом их потомства.

В качестве примера успешного использования ДЭН можно привести недавнее обследование в Армении, проведенное ГСЙ в рамках проекта по разработке низкозатратной методики оценки йодного статуса. В исследование были включены 280 беременных женщин первого триместра из семи дозорных участков, расположенных в пяти целенаправленно избранных районах страны. Участницы рекрутировались во время плановых визитов в выбранные учреждения антенатальной помощи (женские консультации). Медицинский персонал женских консультаций осуществлял отбор образцов мочи при первом обращении женщины за консультацией с последующим аликвотированием приблизительно 5 мл каждого образца в индивидуально маркированные контейнеры. Для каждой участницы заполнялась краткая сопроводительная форма. Все образцы мочи хранились в замороженном виде в соответствующих учреждениях до их транспортировки в Национальный институт здравоохранения в Ереване. Определение концентации йода в моче было выполнено в лаборатории I’MUNO (Тбилиси, Грузия).

По результатам лабораторного анализа образцов мочи 280 беременных женщин первого триместра, обследованных в пяти районах Армении в 2023 году (данные трех дозорных участков города Еревана были объединены), мКЙМ составила 175,3 мкг/л, что свидетельствует об устойчиво адекватном йодном статусе данной группы населения. За исключением относительно более высокого значения мКЙМ (267,5 мкг/л) в одном из районов, уровни мКЙМ среди беременных женщин в остальных дозорных участках были сопоставимы и соответствовали критериям адекватной йодной обеспеченности. В целом полученные данные подтверждают адекватный йодный статус беременных женщин и, по всей вероятности, всего населения Армении в целом. Существенным преимуществом ДЭН является более низкая стоимость обследования, которая в случае Армении составляла приблизительно одну десятую часть затрат, необходимых для проведения национального репрезентативного поперечного обследования. Это делает методологию перспективным инструментом регулярного мониторинга программ йодирования соли и позволяет рекомендовать её дальнейшее применение на постоянной основе даже в условиях ограниченного финансирования [19].

Авторы руководства также рекомендуют изыскивать возможности для оценки йодного статуса путем интеграции исследований КЙМ в более широкие исследования или системы наблюдения. Например, определение КЙМ можно добавить к любой системе обследований, в рамках которых уже собираются образцы мочи (например, для оценки потребления натрия/соли). Аналогичным образом, сбор образцов мочи можно относительно легко добавить к обследованиям, в рамках которых собираются образцы крови. Интеграция оценки йодного статуса в более широкие системы оценки здоровья или питания позволяет эффективнее использовать ресурсы, повышает доступность данных о йодном статусе и облегчает интеграцию мер по улучшению обеспеченности питания йодом в более широкие стратегии и подходы в области здравоохранения и питания.

При оценке йодного статуса приоритет должен отдаваться обследованиям беременных и женщин репродуктивного возраста. Оценку йодного статуса у беременных женщин можно проводить в рамках обследования в женских консультациях (центрах дородового наблюдения). Сбор образцов мочи в женских консультациях при рутинном обращении в них беременных женщин, вероятно, будет менее ресурсоемким процессом, чем репрезентативные поперечные обследования йодного статуса. При обследовании домохозяйств оценку йодного статуса наиболее целесообразно проводить у женщин репродуктивного возраста, которые являются наиболее репрезентативной группой населения, поскольку адекватный йодный статус в этой группе предполагает, что беременность у них также будет протекать на фоне достаточного потребления йода. Однако, поскольку программы обогащения соли направлены на охват всего населения, дети школьного возраста по-прежнему представляют собой ценную альтернативную группу населения, которую можно относительно легко охватить посредством обследований на уровне школ и данные о йодном статусе у которых часто, но не всегда, являются репрезентативными для всего населением.

## Аналитическая записка №6: Использование соли для обогащения другими микронутриентами

Обогащение соли йодом эффективно и устойчиво снизило дефицит йода во всем мире, и поэтому признано одним из самых успешных мероприятий в области общественного здравоохранения за последние несколько десятилетий. Обязательное йодирование соли проводят больше стран, чем обогащение любых других пищевых продуктов или приправ, и большая доля населения мира использует йодированную соль, чем любые другие обогащенные продукты. Успех соли, обогащенной йодом, во многом объясняется преимуществами соли как субстрата обогащения и низкой массовой долей йода, требуемой для обогащения. К преимуществам соли относятся:

В настоящее время из 126 страна мира, в которых действует законодательство об обязательном обогащении соли йодом, в 15 странах разрешено добавлять в соль дополнительный микронутриент: в 14 стран в соль можно добавлять фтор, а Индия — соли железа. Кроме того, в ряде стран в настоящее время исследуют эффективность добавки в соль других микронутриентов (фолиевой кислоты, витамина В12 и цинка) [21].

Глобальная сеть по йоду в 2019 году проведа всесторонний анализа данных о соли, обогащённой йодом и железом. Было установлено, что соль, обогащенная как йодом, так и железом, в условиях пилотных испытаний была способна улучшить потребление железа, особенно при его высоком содержании в соли. Однако в реальных условиях не было получено доказательств улучшения потребления железа и снижения его дефицита у населения. Кроме того, обогащение йодированной соли железом может вызвать органолептические изменения, неприемлемые для потребителей, и привести к потерям йода. Соль — довольно реакционноспособное соединение и гигроскопична, а из-за её способности поглощать воду могут возникнуть проблемы с сохранностью йода. По мнению ГСЙ добавка солей железа в йодированную соль нецелесообразна в силу отсутствия эффективности и отрицательного влияния на эффективность и привлекательность обогащенной йодом соли.

## Заключение

За первую четверть ХХI века соль, обогащенная йодом, стала доступна практически повсеместно, и в мире сохраняются лишь отдельные очаги йодного дефицита. К 2024 году 126 стран мира ввели обязательное обогащение соли йодом, а еще 22 страны разрешают йодировать соль на добровольной основе. В результате этих усилий во всем мире 89% людей потребляют йодированную соль, а из 127 стран, имеющих национальные данные, 101 страна имеет адекватный йодный статус населения и только 18 – недостаточный, тогда как в 1993 г. низкое потребление йода имело место в 113 странах [5]. Именно поэтому обогащение соли йодом признано одним из величайших достижений общественного здравоохранения.

Выпуск новых рекомендаций, подготовленные под эгидой ЮНИСЕФ, обусловлен необходимость в обновлении существующих руководств, регламентирующих обогащение соли, и использовании новых подходов к обогащению соли в сочетании с сохранением и укреплением мер, доказавших свою эффективность и устойчивость.

Из 6 «аналитических записок», сходящих в руководство, наибольший интерес для читателей может предствлять раздел, посвященный повышению доступности данных о йодном статусе населения. По мнению авторов, оценка йодного статуса должна осуществляться путем интеграции обследований йодного статуса в более комплексные обследования здоровья и питания населения либо посредством дозорного эпидемиологического мониторинга в районах высокого риска йодного дефицита. Сбор образцов мочи в женских консультациях при рутинно обращении в них беременных женщин, вероятно, будет менее ресурсоемким процессом, чем репрезентативные поперечные обследования йодного статуса.

## Дополнительная информация

Источники финансирования. Работа выполнена по инициативе автора без привлечения финансирования.

Конфликт интересов. Автор декларирует отсутствие явных и потенциальных конфликтов интересов, связанных с содержанием настоящей статьи.

Благодарности. Автор выражает благодарность Глобальной сети по йоду за поддержку перевода текста «Руководства по обогащению соли йодом: подтвержденная приверженность достижению оптимального уровня йода в питании» на русский язык.

1. До 2010 г. — Международной совет по профилактике йододефицитных заболеваний, ICCIDD.2. В англоязычной научной и технической литератере понятие «salt (food) fortification» обычно переводится на русский язык как «обогащение» соли (или других пищевых продуктов). Термин «фортификация», относящийся к строительству крепостей и других оборонительных сооружений, как синоним «обогащения» соли или иных пищевых продуктов использовать не следует.3. Под термином «пищевые (продовольственные) системы» (food systems) подразумевается вся совокупность процессов производства, переработки, распределения, торговли и потребления пищевых продуктов.4. Определение содержания солей железа, фолиевой кислоты и других микронутриентов, добавляемых в муку в процессе ее обогащения, требует использования сложного и дорогого лабраторного оборудования, и осуществляется специализированными лабораториями. В то же время методы определения йода в соли, такие как йодометрическое титрование, вполне доступны для производственных лабораторий соляных фабрик.5. В ВОЗ входят 194 государства-члена. См.: https://www.who.int/about/who-we-are.6. Обычно аналогом термина «sentinel surveillance» в официальных документах и научных публикациях на русском языке является «дозорный эпидемиологический надзор» (ДЭН).7. Потребление соли в западной части Тихоокеанского региона, вероятно, существенно зависит от Китая, где расчетное потребление соли составляет 17,7 г/сутки. Высоким потребление соли является также в странах Восточной Европы, Южного Кавказа и Центральной Азии.
